# Impact of progesterone concentration on human chorionic gonadotropin trigger day on clinical outcomes with one top-quality cleavage-stage embryo or blastocyst transfer in fresh *in vitro* fertilization cycles

**DOI:** 10.3389/fendo.2023.1085287

**Published:** 2023-06-20

**Authors:** Jianing Xu, Cuilian Zhang, Shuna Wang, Shaodi Zhang

**Affiliations:** Reproductive Medicine Center, Henan Provincial People’s Hospital, People’s Hospital of Zhengzhou University, Zhengzhou, China

**Keywords:** elevated progesterone, GnRH antagonist, fresh cycle, IVF, clinical pregnancy

## Abstract

**Objective:**

To investigate the impact of the progesterone concentration on the human chorionic gonadotropin (hCG) trigger day on clinical outcomes with an antagonist protocol.

**Methods:**

The retrospective cohort study included a total of 1,550 fresh autologous ART cycles with one top-quality embryo transfer. Multivariate regression analysis, curve fitting, and threshold effect analysis were performed.

**Results:**

A significant association was found between the progesterone concentration and clinical pregnancy rate (adjusted OR, 0.77; 95% CI, 0.62–0.97; P = 0.0234), especially in blastocyst transfer (adjusted OR, 0.56; 95% CI, 0.39–0.78; P = 0.0008). The association between the progesterone concentration and the ongoing pregnancy rate was insignificant. The clinical pregnancy rate showed a linear relationship with an increased progesterone concentration in cleavage-stage embryo transfer. In blastocyst transfer, as the progesterone concentration increased, the clinical and ongoing pregnancy rates showed a parabolic reverse-U curve; the curve initially increased before declining at high progesterone concentrations. The clinical pregnancy rate increased with a progesterone concentration up to 0.80 ng/mL rather than tended to be stable. The clinical pregnancy rate significantly decreased when the progesterone concentration was ≥0.80 ng/mL.

**Conclusion:**

The progesterone concentration on the hCG trigger day exhibits a curvilinear relationship with pregnancy outcomes in blastocyst transfer cycles, and the optimal threshold of the progesterone concentration is 0.80 ng/mL.

## Introduction

Serum progesterone (P) elevation during the follicular phase of ovarian stimulation for *in vitro* fertilization (IVF) and its effect on endometrial receptivity have been matters of intense debate and research during the last few decades. Despite the effective suppression of premature luteinization by gonadotropin-releasing hormone (GnRH) analogues, early rises in the P concentration continue to occur in 5% to 38% of all down-regulated assisted reproductive technology (ART) cycles (1, 2). Factors associated with premature elevations in the P concentration include higher doses of exogenous follicle-stimulating hormone (FSH), a higher serum estradiol (E2) concentration on the hCG trigger day, and higher numbers of developing follicles (1, 2).

The negative effects of an elevated P concentration on the day of ovulation trigger during IVF cycles have been well documented. These effects include not only impairment of endometrial receptivity but also detrimental effects on embryo quality (3–5). However, other studies have shown contradictory results indicating that elevated P concentrations are not detrimental to pregnancy outcomes or euploidy rates (6–8). Most studies used an absolute P concentration of 0.8 to 2.0 ng/mL on the day of human chorionic gonadotropin (hCG) administration (1, 9). The different cut-off levels of premature P elevation could have contributed to the controversial conclusions of these studies.

On the fifth or sixth luteal day, the endometrium allows for normal embryo–endometrium cross-talk and implantation potential when the embryo develops into the blastocyst stage. However, most published data regarding the effect of elevated P concentrations on ART outcomes focused mainly on cleavage-stage embryo transfers. Fewer studies focused on the differences in the effects of elevated P concentrations on pregnancy outcomes between different embryo types, and the results were not homogeneous. The present study was performed to determine the impact of the serum P concentration on clinical outcomes such as clinical pregnancy and ongoing pregnancy rate with one top-quality cleavage-stage embryo or blastocyst in antagonist cycles.

## Materials and methods

### Patient data collection

A retrospective, observational, single-center cohort study was conducted. The study was reviewed by the Reproductive Medicine Ethics Committee of Henan Provincial People’s Hospital (SYSZ-LL-2019110401). The inclusion criteria were treatment by IVF and intracytoplasmic sperm injection(ICSI) from 1 January 2014 to 31 December 2021, performance of controlled ovarian stimulation with an antagonist protocol, and a performance of a fresh transfer cycle with one top-quality embryo in the cleavage stage or blastocyst stage. The exclusion criteria were a chromosomal abnormality in either partner; uterine malformations; intrauterine conditions affecting the pregnancy outcomes of embryo transfer, such as endometrial polyps, uterine cavity adhesion, a history of endometrial tuberculosis, or hydrosalpinx with reflux into the uterine cavity; and cycles that underwent pre-implantation genetic testing(PGT). After the corresponding standard screening, 1,550 eligible fresh transfer cycles were included in the study.

### Ovarian stimulation, cycle monitoring, and final oocyte maturation triggering

Ovarian stimulation was initiated on days 2 to 3 of the menstrual cycle with 150 to 300 IU/day of recombinant FSH (Gonal-f; Merck Serono, Darmstadt, Germany). Pituitary down-regulation was performed with daily administration of a GnRH antagonist in a flexible protocol (cetrorelix [Cetrotide]; Merck Serono) starting from the detection of at least one follicle of 14 to 15 mm or a dominant follicle diameter of 12 mm and serum E2 concentration of >300 ng/L.

Cycles were monitored through serial vaginal ultrasound scans and measurement of the serum concentrations of luteinizing hormone(LH), E2, and P. As soon as three follicles of ≥17 mm or two follicles of ≥18 mm were observed, final oocyte maturation was triggered with 5000 or 10,000 IU of urinary hCG and, in patients with a risk of ovarian hyperstimulation syndrome, the addition of 0.2 mg of a GnRH agonist (Decapeptyl; Ipsen, Paris, France). Follicular aspiration was performed 34 to 36 hours after ovulation trigger.

### Embryo transfer and embryo scoring

Before embryo transfer, each embryo was graded according to its developmental speed, degree of fragmentation, and evenness of the cleavage sphere. Embryos with seven to nine blastomeres, uniform cytoplasm, regular morphology, and a fragmentation rate of <10% were considered top-quality embryos. Blastocyst was performed by following the Gardner scoring system, and embryos graded 4BB or better were defined as top-quality embryos.

The decision regarding the optimal embryonic type for transplantation mainly depended on the embryo morphology, patient’s condition, or embryologists’ and clinicians’ suggestions. If more than four to six embryos were present in the cleavage stage, we preferred to follow up the extended culture, which allowed us to choose a higher-quality blastocyst to transfer. If fewer than two to four embryos were present, we generally decided to transfer the cleavage-stage embryos. To avoid the influence of different embryo qualities on pregnancy outcomes, we only selected fresh transfer cycles with one top-quality cleavage-stage embryo or blastocyst in this study.

### Outcome measurement

The serum hCG concentration was measured 14 days after an embryo transfer was conducted to assess the outcome. The dose of estrogen and P remained the same until 14 days after embryo transfer, was gradually reduced after the fetal heart examination, and was completely stopped at gestational week 10. If the serum hCG measurement was positive, an ultrasound examination was performed 2 to 3 weeks later to confirm intrauterine pregnancy and determine the number of gestational sacs. Clinical pregnancy was defined by at least one gestational sac on ultrasonography 4 to 6 weeks after an embryo transfer. Ongoing pregnancy was defined as viable intrauterine pregnancy of at least 12 weeks’ duration confirmed on an ultrasound scan.

The following equations were used to calculate the clinical and ongoing pregnancy rates.

* Clinical pregnancy rate = number of cycles with clinical pregnancy/number of transfer cycles × 100%
* Ongoing pregnancy rate = number of cycles with ongoing pregnancy/number of transfer cycles × 100%

### Progesterone assessment immunoassay

The serum P concentration was assessed on the day of hCG administration using a validated electrochemiluminescence immunoassay (Cobas 8000; Roche, Basel, Switzerland). The lower limit of detection for the assay was 0.05 ng/mL, and the coefficient of variation was 3.3%. The same assay was performed throughout the full duration of the study and was regularly calibrated to minimize variation of the results associated with time and reagent batch renewal.

### Statistical analysis

Statistical analysis was performed using Empower Stats software based on the R language. Continuous variables are presented as mean ± standard deviation or median (interquartile range), and categorical variables are presented as number (frequency). The t test and Kruskal-Wallis test should be used to compare normally distributed and non-normally distributed continuous variables.The chi-square test were used to compare differences in the rates of clinical outcomes. Multivariate regression analysis, curve fitting, and threshold effect analysis were performed on all cycles. Confounders were selected based on their association with the outcomes of interest or a 10% change in the effect estimate. Smooth curve fitting was performed to identify any non-linear relationship between the P concentration and pregnancy outcomes. A piece-wise linear regression method was used to analyze the threshold effect between the P concentration and pregnancy outcomes. Statistical significance was defined as a P-value of <0.05.

## Results

### Baseline and patient characteristics

1,550 cycles with one top-quality embryo transfer were analyzed for the study population. The baseline characteristics included female age; body mass index; duration of infertility; fertilization type; infertility type; basal FSH concentration; basal E2 concentration; basal P concentration; antral follicle count (AFC); gonadotropin (Gn) dosage; Gn duration; E2 concentration, luteinizing hormone concentration, and P concentration on the hCG trigger day; endometrial thickness (EMT); the number of oocytes retrieved; the number of two pronuclear stage (2PN) embryos and embryo type. The overall clinical pregnancy rate per embryo transfer cycle was 46.65% (723/1550), and the overall ongoing pregnancy rate per embryo transfer cycle was 38.58% (598/1550). The patient demographics and IVF/intracytoplasmic sperm injection characteristics are summarized in **Table 1**.

**Table 1:** Characteristics of patients undergoing fresh cycles with one top quality embryo transfer.

Item	Mean (SD)/Median (Q1-Q3)	N (%)
Female age	33.19 (5.12)	
BMI	23.06 (3.70)	
Duration of infertility(year)	3.00 (1.50-5.00)	
Fertilization type		
IVF		1129 (76.18%)
ICSI, rescue-ICSI		336 (22.67%)
IVF & rescue-ICSI		17 (1.15%)
Infertility type		
Primary Infertility		986 (63.61%)
Secondary Infertility		564 (36.39%)
Basal FSH concentration (mIU/ml)	7.22 (5.91-9.16)	
Basal E2 concentration(ng/ml)	39.62 (30.00-54.00)	
Basal P concentration(ng/ml)	0.36 (0.20-0.62)	
AFC	8.00 (5.00-12.00)	
Gn dosage (IU)	2261.40 (822.02)	
Gn duration(day)	9.05 (2.09)	
E2 concentration on hCG trigger day(pg/ml)	1360.00 (822.00-2295.00)	
LH concentration on hCG trigger day(mIU/ml)	2.48 (1.53-3.79)	
P concentration on hCG trigger day(ng/ml)	0.73 (0.40-1.11)	
EMT on hCG trigger day (mm)	10.57 (2.27)	
No. of retrieved oocytes	6.00 (4.00-10.00)	
No. of 2PN embryo	4.00 (2.00-6.00)	
Embryo type		
cleavage-stage embryo		949 (61.23%)
blastocyst		601 (38.77%)
Clinical pregnancy in the fresh cycle(;%)		723 (46.65%)
Ongoing pregnancy in the fresh cycle(;%)		598 (38.58%)

body mass index (BMI); antral follicle counting (AFC); gonadotropin (Gn); estradiol(E2), luteinizing hormone (LH), progesterone(P); endometrium thickness (EMT).

### Univariate analysis of factors associated with clinical outcomes

Univariate analysis was performed to identify the indicators that affect clinical pregnancy and ongoing pregnancy outcomes with one top-quality embryo transfer. Female age had a negative influence on the clinical pregnancy rate and ongoing pregnancy rate (P < 0.05). Infertility type, AFC, Gn duration, EMT, number of retrieved oocytes, and number of 2PN embryos were associated with an increased clinical pregnancy rate (P < 0.0001). In blastocyst transfer, the clinical pregnancy rate and ongoing pregnancy rate were also significantly increased. (P < 0.0001). Detailed data are shown in **Table 2**.

**Table 2:** Univariate analysis of factors associated with pregnancy outcomes.

	Clinical pregnancy		Ongoing pregnancy	
	OR (95%CI)	P	OR (95%CI)	P
Female age	0.92 (0.90, 0.94)	<0.0001	0.91 (0.89, 0.93	<0.0001
Fertilization type				
IVF	1		1	
ICSI, Rescue-ICSI	0.91 (0.71, 1.16)	0.4578	0.93 (0.72, 1.19	0.5509
IVF & Rescue-ICSI	1.61 (0.61, 4.27)	0.3345	1.78 (0.68, 4.65	0.2384
BMI	1.01 (0.98, 1.04)	0.5275	1.02 (0.99, 1.04	0.2472
Infertility type				
Secondary Infertility	1		1	
Primary Infertility	1.41 (1.15, 1.74)	0.0011	1.44 (1.17, 1.78	0.0007
Duration of infertility(year)	0.97 (0.94, 1.00)	0.0689	0.97 (0.94, 1.01	0.1204
Basal FSH (mIU/ml)	0.99 (0.97, 1.02)	0.6515	0.98 (0.96, 1.01	0.1776
Basal E2 (ng/ml)	1.00 (0.99, 1.00)	0.478	1.00 (0.99, 1.00	0.2904
Basal P (ng/ml)	1.05 (0.83, 1.31)	0.7009	1.12 (0.89, 1.42	0.3187
AFC	1.03 (1.01, 1.04)	0.0059	1.02 (1.01, 1.04	0.0119
Gn dosage (IU)	1.00 (1.00, 1.00)	0.4003	1.00 (1.00, 1.00	0.2303
Gn duration (day)	1.08 (1.03, 1.13)	0.0029	1.10 (1.05, 1.16	0.0001
E2 level on hCG trigger day(pg/ml)	1.00 (1.00, 1.00)	0.1146	1.00 (1.00, 1.00	0.0748
LH level on hCG trigger day(mIU/ml)	1.00 (0.96, 1.03)	0.8449	0.98 (0.94, 1.02	0.3659
P level on hCG trigger day(ng/ml)	1.05 (0.86, 1.28)	0.6207	1.19 (0.97, 1.46	0.0946
EMT on hCG trigger day (mm)	1.09 (1.04, 1.14)	0.0001	1.08 (1.03, 1.13	0.0007
No. of retrieved oocytes	1.02 (1.00, 1.05)	0.0311	1.03 (1.01, 1.05	0.0136
No. of 2PN embryo	1.04 (1.00, 1.07)	0.0314	1.05 (1.01, 1.08	0.005
Embryo type				
cleavage-stage embryo	1		1	
blastocyst	1.97 (1.60, 2.42)	<0.0001	1.99 (1.61, 2.45	<0.0001

body mass index (BMI); antral follicle counting (AFC); gonadotropin (Gn); estradiol(E2), luteinizing hormone (LH), progesterone(P); endometrium thickness (EMT).

### Associations between P concentration and clinical outcomes of fresh cycles using multivariable logistic regression analysis

Confounders were selected based on their association with the outcomes of interest or a 10% change in the effect estimate. Multivariable regression analysis was performed by adjusting for selected confounders such as female age, BMI, Gn duration, EMT on the hCG trigger day, infertility type, AFC, and number of retrieved oocytes. A significant associations was found between the P concentration and clinical pregnancy rate (adjusted odds ratio [aOR], 0.77; 95% confidence interval [CI], 0.62–0.97; P = 0.0234), especially in blastocyst transfer (aOR, 0.56; 95% CI, 0.39–0.78; P = 0.0008). The association between the P concentration and ongoing pregnancy rate was not statistically significant (**Table 3**).

**Table 3:** Associations between P and clinical outcomes of fresh cycles using multivariable logistic regression analysis.

Outcome	Clinical pregnancy		Ongoing pregnancy	
	OR (95%CI)	P	OR (95%CI)	P
Cleavage stage embryo	1.03 (0.76, 1.40)	0.8470	1.20 (0.88, 1.65)	0.2542
Blastocyst	0.56 (0.39, 0.78)	0.0008	0.67 (0.48, 0.95)	0.0224
Total	0.77 (0.62, 0.97)	0.0234	0.91 (0.97, 1.14)	0.4200

Adjusted for female age; Gn duration; EMT on HCG trigger day; Infertility type; AFC; No. of retrieved oocytes; BMI.

### Curve fitting between EMT and clinical outcomes in different embryo types

**Figure 1:**
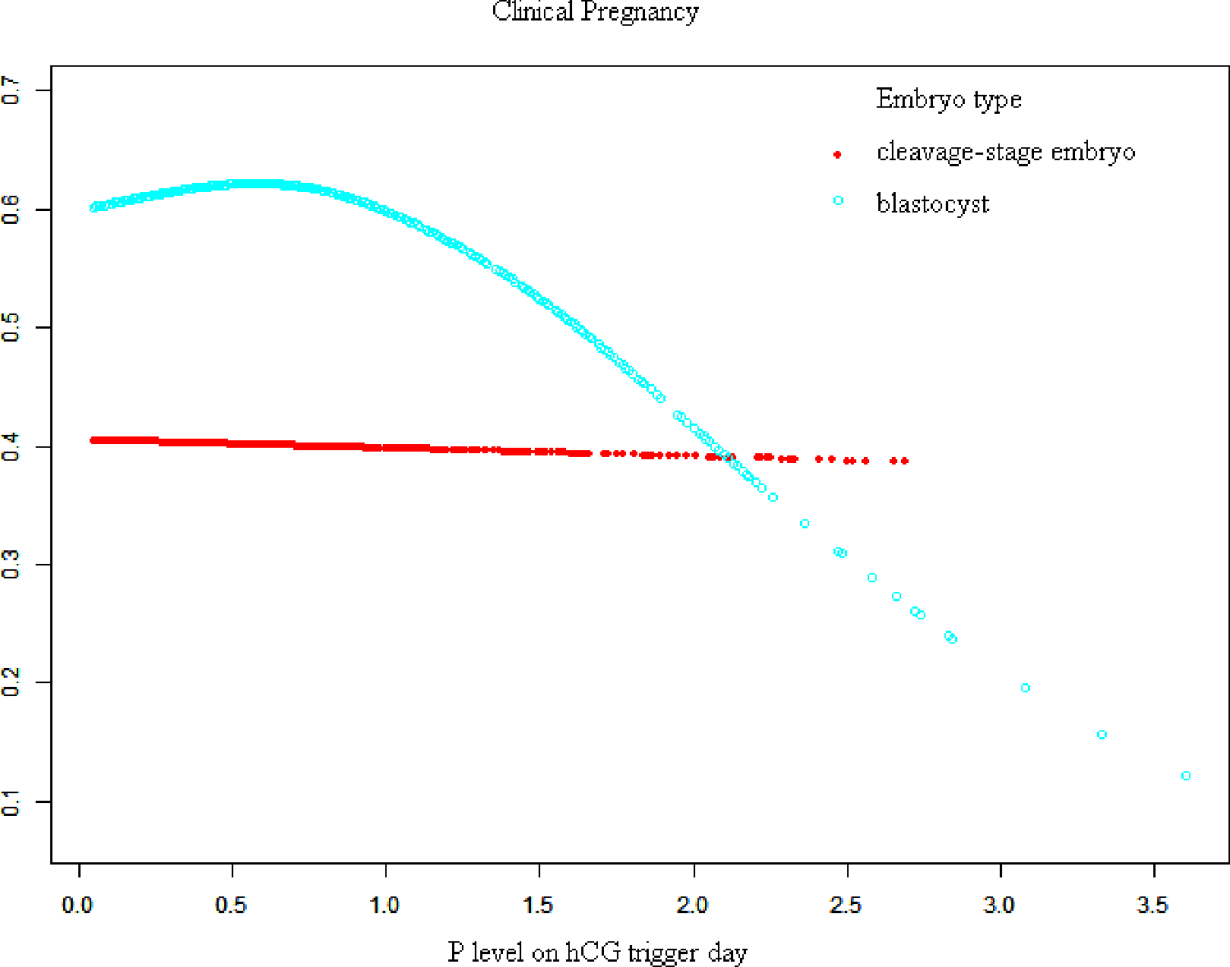
Association between progesterone concentration and clinical pregnancy rate in different embryo types. A threshold, nonlinear association between the progesterone concentration and clinical pregnancy rate was found in blastocyst transfer in a generalized additive model. The fitted line was adjusted for female age, BMI, gonadotropin duration, endometrial thickness on the human chorionic gonadotropin trigger day, infertility type, antral follicle count, and the number of retrieved oocytes.

**Figure 2:**
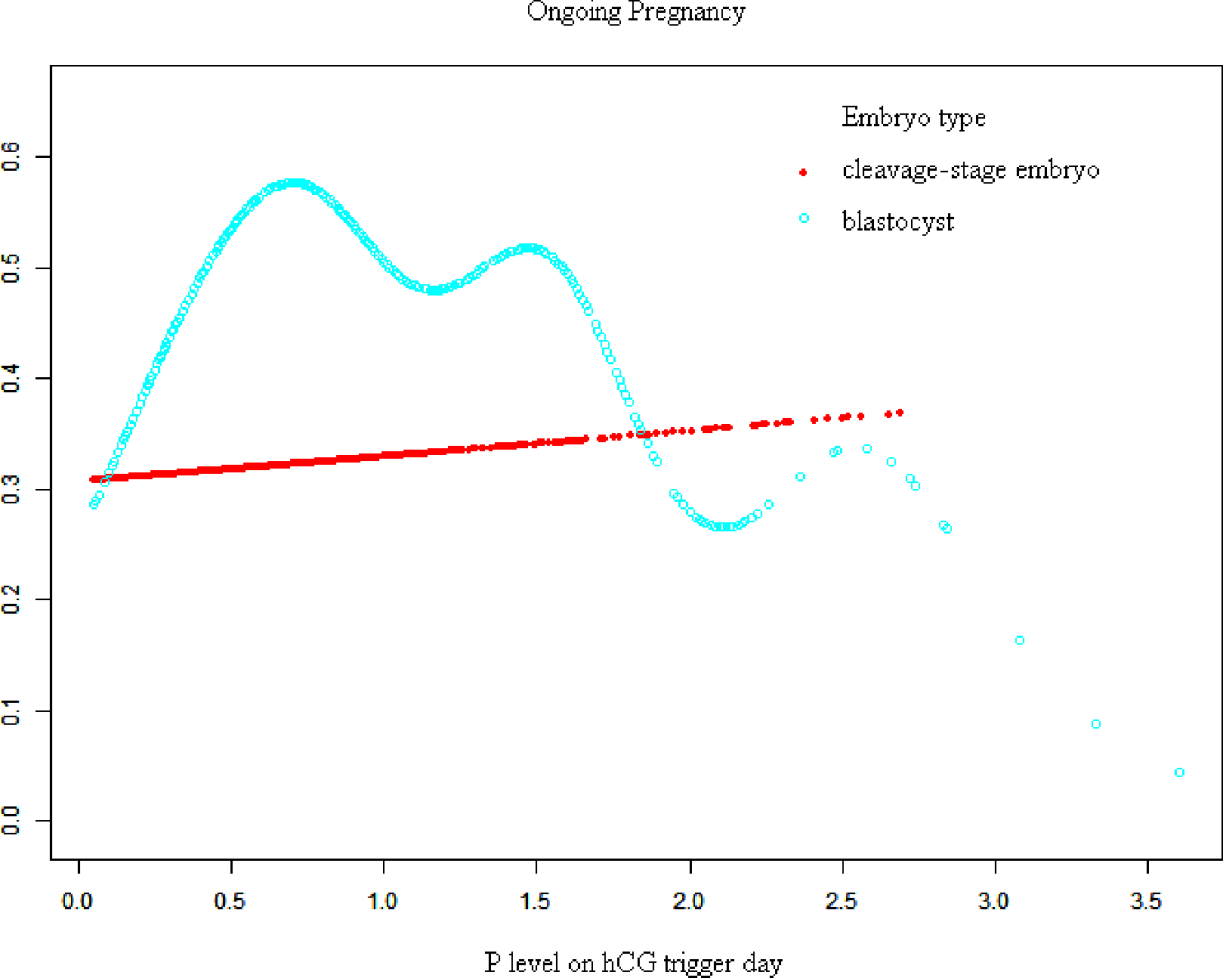
Association between progesterone concentration and early miscarriage rate in different embryo types. A threshold, nonlinear association between the progesterone concentration and ongoing pregnancy rate was found in blastocyst transfer in a generalized additive model. The fitted line was adjusted for female age; BMI, gonadotropin duration, endometrial thickness on the human chorionic gonadotropin trigger day, infertility type, antral follicle count, and the number of retrieved oocytes.

**Figures 1**, **2** show fitted curves with adjustment for confounders showing the relationship between the P concentration and clinical outcomes. The clinical pregnancy rate showed a linear relationship with an increased P concentration in cleavage-stage embryo transfer. In blastocyst transfer, the clinical and ongoing pregnancy rates showed a parabolic reverse-U curve as the P concentration increased. The curve initially increased before declining at high P concentrations.

### Threshold effect analysis of P concentration and clinical outcomes in fresh cycles using the piece-wise linear regression method

When transferred with blastocyst, there is a threshold of these curves. The results of the threshold effect analysis of the P concentration and the clinical outcomes are presented in **Table 4**. The clinical pregnancy rate increased with the P concentration up to 0.80 ng/mL. The clinical pregnancy rate significantly decreased when the P concentration was ≥0.80 ng/mL,

**Table 4:** Threshold effect analysis of progesterone concentration and clinical outcomes in fresh cycles using the piece-wise linear regression method.

Blastocyst	Cutoff of P level	Effect size (OR)	95%CI	P
Clinical pregnancy (CPR)	< 0.80	1.66	0.59, 4.69	0.3415
	≥0.80	0.40	0.25, 0.64	0.0001
Ongoing pregnancy (OPR)	< 0.80	2.68	0.95, 7.58	0.0624
	≥0.80	0.44	0.28, 0.70	0.0006

Adjusted for female age; Gn duration; EMT on hCG trigger day; Infertility type; AFC; No. of retrieved oocytes; BMI.

## Discussion

To the best of our knowledge, this is the largest investigation to demonstrate the relationship between the P concentration on the day of hCG administration and pregnancy outcomes such as the clinical pregnancy rate and ongoing pregnancy rate using a GnRH antagonist protocol with one top-quality embryo transfer, avoiding the bias of embryo number and quality. Our data confirm the findings of previous studies showing a detrimental association of elevated serum P concentrations on the day of hCG administration with clinical pregnancy (10, 11). Our data also demonstrated that this detrimental impact on clinical pregnancy occurred especially in top-quality blastocyst-stage transfers, rather than cleavage-stage embryo transfers, using this protocol.

The impact of elevated P concentrations on the pregnancy rate is of utmost importance, and several studies on this topic have been performed. One multicenter, retrospective matched case–control analysis including antagonist cycles with a freeze-all policy indicated that the cumulative live birth rate did not differ between the two study groups (29.3% and 28.2% in the normal P group [P < 1.5 ng/mL] and elevated P group [P > 1.5 ng/mL], respectively; P = 0.773) (6). Connell et al. (12) examined whether the P concentration on the day of trigger had similar effects on pregnancy outcomes in hCG and GnRH agonist trigger IVF cycles. They evaluated outcomes in cycles with a P concentration of ≥2 and <2 ng/mL and found that a P concentration of ≥2 ng/mL had a similar negative effect on live birth in both cohorts. In addition, the data obtained by Oktem et al. (13) suggested that a rise in the serum P concentration from <0.5 to >4.0 ng/mL caused a gradual and continuous decline in the clinical pregnancy rate of different types of ovarian responses; even high responders were not exempt from the detrimental effects of prematurely rising serum P concentrations. These variable conclusions may be due to the different cut-offs selected among the studies. However, another large meta-analysis evaluated the association of P elevation on the day of hCG administration with the probability of pregnancy in fresh IVF cycles based on more than 60,000 cycles. The results of eligible studies were combined for the meta-analysis using the inverse variance method and meta-regression analysis. The data indicated that P elevation on the day of hCG administration was significantly associated with a decreased achievement of pregnancy from P levels of >0.8 ng/mL (the pooled effect sizes were 0.8–1.1 ng/mL: OR = 0.79, 1.2–1.4 ng/mL: OR = 0.67, 1.5–1.75 ng/mL: OR = 0.64, and 1.9–3.0 ng/mL: OR = 0.68 (P < 0.05 in all cases) (14). The cut-off value from this survey is similar to ours, but the embryo type, quality, and cycles were not homogenized. We also found an approximate reverse-U-shaped association between the P concentration and ongoing pregnancy rate in blastocyst transfer cycles. Despite a better understanding of the association between elevated P concentrations and pregnancy rates, there are little data to evaluate the effect of decreased P concentrations on pregnancy outcomes in ART cycles. In a study by Arvis et al. (15), the P concentration was combined with a strong quadratic effect (OR = 0.585 per Log (2) (phCG) ng/mL, 95% CI = 0.444–0.775, P < 0.001), resulting in a reverse-U curve. The authors concluded that P concentrations of ≤0.5 ng/mL were as detrimental to live birth as high P concentrations, which may be due to decreased luteinization, altered endometrial receptivity, or both.

Despite numerous findings of the effects of elevated P concentrations on clinical outcomes in every aspect, such analyses in regard to different types or days of embryo transfer are rare and controversial. Huang et al. (9) reported a detrimental effect on day 5 blastocyst-stage transfer only when the P concentration reached 1.75 ng/mL. In another observational cohort study, Tokgoz and Tekin (10) evaluated the effect of an elevated P concentration on the day of hCG administration in patients stimulated with a GnRH antagonist protocol. Day-3 embryo transfers with a P concentration of >0.85 ng/mL led to a relative reduction of >20% in clinical pregnancy (aOR, 0.424; P = 0.016). However, day-5 embryo transfers led to similar clinical pregnancy rates regardless of whether the P concentration was elevated or normal. Hill et al. (1) reported that elevated P concentrations on the day of the hCG trigger were associated with a declining linear association with live birth for both cleavage-stage and blastocyst-stage transfers. Our data demonstrated a significant association between the P concentration and clinical pregnancy rate (aOR, 0.77; 95% CI, 0.62–0.97; P = 0.0234). It seems that the critical threshold for a detrimental effect differs depending on whether embryo transfer is performed at the blastocyst stage or cleavage stage (aOR, 0.56; 95% CI, 0.39–0.78; P = 0.0008). A curve-fitting and threshold analysis further revealed a quantitative relationship between the P concentration and clinical outcomes with different embryo types. There was a linear relationship between the P concentration and clinical pregnancy in the cleavage-stage embryo transfer cycle. In blastocyst transfer, however, the clinical pregnancy and ongoing pregnancy rates decreased sharply when the P concentration was ≥0.80 ng/mL (clinical pregnancy rate: aOR, 0.40; 95% CI, 0.25–0.64; P = 0.0001 and ongoing pregnancy rate: aOR, 0.44; 95% CI, 0.28–0.70; P = 0.0006). Possible explanations for the discrepancy between these studies include different sample sizes, differences in ovarian stimulation protocols (with or without an agonist protocol), differences in laboratory culture conditions, and differences between the P assays used.

Histologic endometrial advancement has been demonstrated to occur in patients undergoing ART on the day of oocyte retrieval (16). Endometrial receptivity depends on the duration of P exposure. The effect of the P concentration on endometrial decidualization is paramount in successful embryo implantation. Early elevations in the P concentration have been proposed to lead to asynchronous development between the endometrium and developing embryo, decreasing the possibility of implantation (17). An altered epigenetic modification status or gene expression in the endometrium with a high P concentration may also disrupt the endometrial receptivity and lead to reduced pregnancy rates (3, 18). Therefore, one premise of this phenomenon lies within the ability of an elevated P concentration to advance the endometrium too quickly, leading to a narrowing in the window of implantation for the embryo (19). Prolonged elevation of the P concentration may cause an inappropriately advanced endometrium, leading to early closure of the implantation window and therefore significantly decreased clinical and ongoing pregnancy rates (20). If the embryo is implanted while the window is closing, leading to possible suboptimal invasion of the trophoblastic tissue, it is biologically plausible that the risk of pregnancy failure and miscarriage would increase. Because the blastocyst transfer time is 2 or 3 days later than the cleavage-stage embryo transfer time, we speculate that blastocyst transfer is more likely influenced by narrowing of the implant window, presenting a different association in our study. According to related studies, there has been some suggestion that for patients who adopt the freeze-all strategy with an elevated P concentration, a feasible detection of implantation window could be conducted to verify the consistency between theories and clinical outcomes.

Our study may help to define the proper threshold for fresh embryo transfer of different embryo types. It seems that blastocysts are more sensitive to variations in the serum P concentration than are cleavage-stage embryos. The shape of the clinical pregnancy rate curve in cleavage-stage embryos suggests that elevation of the P concentration may be ignored in such cases, thus avoiding unnecessary cancellations or embryo freezing. Conversely, in blastocyst transfer, the negative effect of P elevation seems to be more pronounced, suggesting that the complete freezing policy should be applied more widely when the P concentration is high, avoiding the waste of top-quality embryos. In blastocyst transfer, the optimal threshold of the P concentration on the day of hCG administration for the fresh cycle is 0.80 ng/mL. Patients in such conditions could benefit from a freeze-all strategy because of the negative effect of a high P concentration (≥0.80 ng/mL).

This study has two main limitations. First, although we reviewed our database with strict inclusion and exclusion criteria, the retrospective design of the study still resulted in some inevitable restrictions, leading to possible overestimation of the results. Second, the observations in our study were based on fresh cycles with an antagonist protocol, and the conclusions may not apply to other IVF stimulated cycles. A well-designed randomized clinical trial will be needed for further study.

## Conclusions

Blastocysts are more sensitive to serum progesterone concentration variations than cleavage-stage embryos. The progesterone concentration on the hCG trigger day exhibits a curvilinear relationship with pregnancy outcomes in blastocyst transfer cycles, and the optimal threshold of the progesterone concentration is 0.80 ng/mL.

## Data availability statement

The original contributions presented in the study are included in the article/**Supplementary Material**. Further inquiries can be directed to the corresponding author.

## Ethics statement

The study was reviewed by the Reproductive Medicine Ethics Committee of Henan Provincial People’s Hospital (SYSZ-LL-2019110401).

## Author contributions

SZ supervised the entire study, including procedures, conception, design, and completion. SW and JX were responsible for collecting information. JX contributed to the analysis data and drafted the manuscript. CZ participated in revising the article. All authors contributed to the article and approved the submitted version.
