# Supplementary material for: Impact of progesterone concentration on human chorionic gonadotropin trigger day on clinical outcomes with one top-quality cleavage-stage embryo or blastocyst transfer in fresh in vitro fertilization cycles
Source: Front Endocrinol (Lausanne). 2023 Jun 20;14:1085287. doi: 10.3389/fendo.2023.1085287 (PMC10319152; doi:10.3389/fendo.2023.1085287)
Supplement: Supplementary file 1 [file Table_1.docx]

Supplement Table 1. Associations between P and clinical outcomes of fresh cycles using multivariable logistic regression analysis.

| Outcome | Early Abortion | |
| --- | --- | --- |
|  | OR (95%CI) | P |
| Cleavage stage embryo | 0.71 (0.37, 1.36) | 0.3017 |
| Blastocyst | 0.76 (0.34, 1.66) | 0.4853 |
| Total | 0.71 (0.43, 1.16) | 0.1717 |

Adjusted for female age; Gn duration; EMT on hCG trigger day; Infertility type; AFC; No. of retrieved oocytes;BMI.
